# The impact and the challenges of implementing a faculty development program on health professions education in a Brazilian Medical School: a case study with mixed methods

**DOI:** 10.1186/s12909-023-04754-8

**Published:** 2023-10-20

**Authors:** Karine Angélica Cintra, Marcos Carvalho Borges, Maria Paula Panúncio-Pinto, Luiz Ernesto de Almeida Troncon, Valdes Roberto Bollela

**Affiliations:** 1 Department of Internal Medicine, Ribeirão Preto Medical School (FMRP-USP), University of São Paulo, Ribeirão Preto, SP Brazil; 2 Department of Internal Medicine & Center for Faculty Development, Ribeirão Preto Medical School (FMRP-USP), University of São Paulo, Ribeirão Preto, SP Brazil; 3 Department of Health Sciences & Center for Faculty Development, Ribeirão Preto School of Medicine (FMRP-USP), University of São Paulo, Ribeirão Preto, SP Brazil

**Keywords:** Faculty development, Teaching–learning, Teaching training, Program evaluation

## Abstract

**Purpose:**

Faculty development in health professions education is still challenging in developing countries like Brazil. Work overload and the lack of financial support hinder faculty members' participation. Ribeirão Preto Medical School founded its Center for Faculty Development in 2016. Since then, an essential skills module (ESMo) on health professions education (HPE) has been offered regularly to faculty members and preceptors of seven undergraduate programs. This case study aims to evaluate the impact of this Essential Skills Module on the educational practices of participants two years after attending the module and the challenges faced during the process.

**Method:**

The study used a mixed-method approach with a description of the demographic and professional profile data of the ESMo participants. Immediate post-ESMo perceptions (satisfaction and learning) of the participants were determined with structured instruments. Two years later, a semi-structured interview was conducted and recorded to determine the long-term effects (application of learning and behavior changing as an educator). *NVIVO®* software was used to store and systematize the thematic discourse analysis with a socio-constructivist theoretical framework interpretation.

**Results:**

One hundred forty-six participants were included: 86 (59%) tenured faculty members, 49 (33,5%) clinical preceptors, and 11 (7,5%) invited teachers. Most were female (66%), and 56% had teaching experience shorter than ten years. 52 (69%) out of 75 eligible participants were interviewed. The immediate reaction to participating in the module was quite positive and 80% have already implemented an educational intervention in their daily activities. Discourses thematic analysis showed five emerging themes appearing in different frequencies: Changes in teaching activities (98%); Lack of previous pedagogical training (92.3%); Commitment and enthusiasm towards teaching (46.15%); Overlapping functions inside the institution (34.6%) and Challenges for student assessment (23%).

**Conclusion:**

This first in-depth evaluation of the long-term effects of a faculty development intervention in a Brazilian Health Profession Education school showed that participation positively changed participants' teaching & learning practices. These interventions consistently fostered a community of practice and valued faculty development processes in local and national scenarios.

## Background

Since 2011, Steinert (2011) has been challenging us to see Faculty Development (FD) more broadly, considering that faculty members' roles have been changing significantly and FD has become an increasingly important component of medical education [1].

FD in health professions education (HPE) remains a challenge for universities and higher education institutions in developing countries, especially in Brazil [2–4]. In 2014, the Ministry of Education published the Brazilian Curricular Guidelines (BCG) for undergraduate medical programs. It stated that schools should *“maintain permanent FD programs to promote and value undergraduate teaching and learning committed with the medical school transformation”* [5]. Despite this recommendation, subsequent changes in the past eight years were limited. Work overload affecting teachers and health professionals, adverse cultural contexts, and the lack of financial investment have been significant obstacles [6, 7], thus contributing to low advancement in this field and negligible scientific production in Brazil [8].

Although mandatory in Brazilian Medical schools, FD programs are still limited in number and actions. In the past two and half decades, the Brazilian Regional Institute for Health Professions Education [8], supported by the Ministry of Health and the Foundation for Advancement of International Medical Education and Research (FAIMER) from Philadelphia, USA, contributed to enhancing the FD scenario in Brazil. FAIMER Brazil has positively affected many HPE institutions and faculty members, supporting them in developing skills in leadership, educational management, teaching methodologies, student assessment, and program evaluation [9]. However, the influence of these initiatives was restricted to a few institutions.

Ribeirão Preto Medical School at the University of São Paulo (FMRP-USP) was founded in 1952 with the support of the USA Rockefeller Foundation [10]. FMRP-USP currently has seven undergraduate courses (Medicine, Biomedical Sciences, Nutrition, Physical Therapy, Speech Therapy, Biomedical Informatics, and Occupational Therapy), 16 departments with 330 Faculty members, and over 1,000 clinical preceptors working in different areas and clinical scenarios. FD initiatives had always been isolated and occasional at FMRP-USP [11, 12].

In 2016, a Center for Faculty Development (CFD) in HPE was founded at FMRP-USP. The primary CFD activity is a structured basic training program entitled Essentials Skills Module (ESMo), which is guided by the best evidence and practices in the HPE field and offered regularly to the faculty community. This case study aims to evaluate the impact of this ESMo on the educational practices of attending Faculty members, both immediately after and two years following the intervention. We also aimed to identify the challenges faced during the CFD implementation process.

## Methods

### Study population and context

All faculty members at FMRP-USP and University Hospital Complex (HCRP) who voluntarily attended one of the five editions of the ESMo were eligible. Those who agreed to participate and signed the informed consent term were included.

The CFD offers ESMo and other short, in-depth workshops on specific HPE themes, such as item writing, team-based learning, flipped classroom, OSCE, workplace-based assessment, Teaching and Learning (T&L) in clinical settings, and interactive lectures, among others. During the COVID-19 pandemic, several synchronous and asynchronous remote learning workshops were designed and delivered intensively by the CFD [13].

From January 2017 to December 2019, the CFD offered five editions of ESMo with 40 places and a five-week duration each. The total workload for participants (30 hours) was divided into 6 hours per week, 4 hours on face-to-face activities and 2 hours on remote learning on a virtual learning environment (VLE) based on the University *Moodle* platform. The ESMo addressed the following themes: Adult Learning Principles; Curriculum design and management; T&L strategies; Student Assessment; and Program Evaluation.

One week before each in-site session, participants were invited to a preparatory forum in Moodle VLE. Before the first in-site session, they were asked to introduce themselves and to read and comment on a short text entitled “*What makes a good teacher?*” [14].

During the ESMo face-to-face sessions, interactive lectures and small group work were the main strategies used and participants were randomly allocated into small groups to stimulate interpersonal interactions. In the first ten minutes, we summarized the main topics discussed in the virtual forum and the previous sessions. This model and structure follow FAIMER Institute USA and FAIMER BRAZIL experiences [15]. At the end of each session, participants fulfilled an open-ended questionnaire regarding what they liked most, what should be changed, and how it would be even better. The ESMo facilitators and CFD faculty analyzed these forms after each session. They always commented on the main points raised and at least one thing that had been changed based on participants' suggestions.

At the final face-to-face session, each participant had four hours to discuss and elaborate a pedagogical intervention proposal to qualify their context practices. The proposal consisted of a three-page file to present what they would like to modify or improve in their teaching practice based on what they already knew or had learned in the ESMo. The proposals should follow a template with these sessions: *a title and a brief description of the teaching/evaluation strategy the participants intended to use in their educational practice; a description of the context, the intervention, and how and when it will be implemented; and how they want to evaluate the intervention based on expected effect*. Participants had 15 days to elaborate and submit their proposal in the *Moodle* VLE and were due to receive written feedback within two weeks.

At the end of the last face-to-face session, a detailed evaluation form was available online for the participants to provide feedback on the entire experience. It was a closed-ended form, based on a retrospective “Pre- & Post-” format, in which each participant indicated their perception about their level of knowledge or competence on each topic addressed on the ESMo, comparing the final to the initial moment [16].

Certification of participation in the ESMo required in-person attendance to at least four of the five face-to-face encounters, plus meaningful involvement in the virtual forums, and, specifically, the submission of the educational intervention proposal. However, its implementation was not formally required. Nevertheless, they were informed about a range of potential outcomes for their proposal such as a successful proposal implementation in a curricular unit or clerkship; a presentation in an education or professional specialty conference; an invitation to teach others this new strategy or approach; and a paper to be submitted to a congress and eventually have it published in a peer-reviewed journal. These outcomes represent measurable achievements and evidence of different levels of scholarship in HPE [4].

The evaluation of the results of the ESMo in HPE followed the Kirkpatrick model [17], which addresses reaction (satisfaction), learning, and real changes and impact on participants and their institutions [18]. Qualitative methods and participant follow-up allowed an in-depth evaluation regarding levels 2 and 3 of the Kirkpatrick pyramid [19–21].

### CFD Educators

The CFD core group includes an educationalist and now (2023) 15 facilitators from all different courses and Departments within the school. Four of us have had links with the FAIMER Institutes (Brazil or USA) [15]. We started in 2017 with seven core members and grew based on invitations made to participants who had an outstanding performance in the ESMo workshops they attended in the first three years. We believe that this is another way to recognize talents and create a centripetal force to grow the FD team in the institution [7, 15].

### Study design and Data collection

We used a mixed-method approach. We described quantitative data from participants, such as sex, professional profile (department, teaching experience duration, academic titles, previous experience with active methodologies), and recorded the main topic addressed in the required pedagogical intervention proposals. We also determined the immediate participant reaction after the course (Kirkpatrick level 1) by analyzing an open-ended form applied after each session. The qualitative approach included faculty participants who concluded ESMo two years before and agreed to participate in the semi-structured interviews conducted personally by the investigator (Kirkpatrick levels 2 and 3). The interviews were previously scheduled and contained 9 open-ended questions (Table 1). They were conducted in person (face-to-face) until the COVID-19 pandemic outbreak and followed the same protocol using Google Meet®. Faculty members who participated in the 2019 ESMo editions were not invited to the interviews because we hypothesized that modifications in the educational scenario due to COVID-19 and the need for emergency remote education could be confounding factors that should be avoided.

**Table 1:** Interview questions to the participants of Essential Skills Module (ESMo)

Year and semester of participation: _________
1) What was your main motivation to participate in the ESMo in HPE?
2) Have you had any administrative difficulty in attending the ESMo meetings? If so, tell us about it. And how did you manage to solve it?
3) Did you have any previous expectations regarding the content you would like to learn in this Faculty Development Program?
4) Did you find the content offered during the ESMo pertinent to your current teaching practice? Please, tell us why
5) Have you already used any of the teaching and learning (T&L) or assessment techniques addressed during the ESMo? If yes: What are they? If not: did you consider using it shortly?
6) Did you implement (partially or totally) the intervention proposal that you made at the end of the ESMo? If yes: in which of the modalities below it might be included? ◦ Curriculum ◦ T&L strategies ◦ Student assessment ◦ Educational program evaluation
7) If not, do you still intend to implement your proposal?
8) If you have already implemented it, did you have the opportunity to evaluate if the implemented proposal improved the educational practice? If so, how did you do that?
9) Did you notice any change in your perception about yourself as a health professions educator after the ESMo? If so, tell us more about it

The study's core question was: *“Did the ESMo in HPE influence changes in your teaching or preceptorship practice?”* All interviews were recorded with the participant's consent, and the audio files were transcribed into a Microsoft Word® document for further content analysis.

### Data analysis

Quantitative data were organized in Excel® files and presented in tables and graphs. Interview transcriptions underwent content analysis after data coding, allowing the identification of recurrent emerging categories in participants' speeches [22–24]. Using a socio-constructivist framework interpretation made it possible to develop a theoretical explanation from the data obtained [18, 19]. The *NVIVO®* software (NVIVO. Release Version 1.6 (1121) 2022c) was used for data storage and analysis systemization. The emerging categories identified from subcategories were associated with excerpts from participants' speeches, enabling an in-depth data analysis and interpretation. The *NVIVO®* consulting tools used were word frequency, encoding, encoding matrix, and cross-reference table.

The study was approved by the Institutional Ethics and Research Committee of the São Paulo University, Ribeirão Preto School of Medicine from the institutional Ethics and Research Committee (Approval n. 02653818.9.0000.5440).

### Participant profile

The study included 146 participants who had completed one of the five ESMo editions, 86 (59%) tenured faculty members, 49 (33,5%) clinical preceptors, and 11 (7,5%) invited teachers. For the qualitative analysis, 82 tenured faculty members with academic department affiliation participated in one of the first three ESMO editions, and 75 met the inclusion criteria for the interview. From this subgroup, 52 (69.3%) agreed to participate. Table 2 shows participants' profiles according to sex, academic degree, years of teaching activity, academic department affiliation in FMRP-USP (clinical medical areas, non-medical clinical areas, basic science areas), other affiliations, and external professionals.

**Table 2:** Participants profile that participated in the Essential Skills Module (ESMo)

PARTICIPANTS	TOTAL	Qualitative Analysis
	**(*n* = 146)**	**(*n* = 52)**
**Gender**		
**Female**	**97 (66%)**	**33 (63,5%)**
**Male**	**49 (34%)**	**19 (36,5%)**
**Academic Degree**		
**Specialist**	**11 (7,5%)**	**1 (2%)**
**Master**	**19 (13%)**	**3 (5,7%)**
**PhD**	**98 (67%)**	**38 (73%)**
**Full/Lecturer Professor**	**18 (12,4%)**	**10 (19,3%)**
**Teaching Time**		
** < 5 years**	**36 (24,8%)**	**15 (28,8%)**
**5–10 year**	**42 (28,7%)**	**11 (21,2%)**
**11–20 years**	**46 (31,5%)**	**17 (32,7%)**
** > 20 years**	**22 (15%)**	**9 (17,3%)**
**Department in FMRP-USP**		
**Basic Areas**	**25 (7,5%)**	**15 (4,5%)**
**Medical Clinical Areas**	**38 (11,5%)**	**22 (6%)**
**Non-Medical Clinical Areas**	**23 (7%)**	**9 (2,7%)**
**Other affiliation**	**49**	**6**
**External professional**	**11**	**0**

## Results

### Previous experience with active methodologies and pedagogical intervention proposals

Among the interviewed participants, 25% reported previous attendance of FD initiatives and the regular use of student-centered T&L methods before the ESMo; 35% reported using student-centered T&L methods, even without any previous training; and 40% reported not using these methods before.

Regarding the themes of the pedagogical proposal they made, 65% addressed themes related to T&L strategies to increase students' engagement and active learning. Other themes, such as student assessment (16%), formative assessment as T&L strategy (12%), and curriculum design (7%) were also identified. Among the T&L strategies proposed, the introduction of the flipped classroom, and interactive and team-based learning, were the most frequent themes. Among the proposals addressing student assessment many intended to initiate a direct observed clinical examination with feedback using standardized patients and in the workplace.

Eighty percent of Faculty members reported that they have already successfully implemented the intervention proposal into their work areas in the two years after completion of ESMo. Half of them reported that the implementation was done at least in part or with some change or adaptation to the original proposal.

### Immediate reactions prompted by the course

The reaction/satisfaction of the participants immediately after the end of the intervention was quite positive for all of them. The main strengths cited were interactive dynamics in small groups; a relaxed and cozy environment; the use of different T&L strategies at each meeting and the remote activities; objectivity, alternation, and motivation of the facilitators in the face-to-face moments; and the possibility to reflect on practice. Strict compliance with the schedule proposed and healthy snacks with fruits were also applauded.

### Interviews qualitative analysis

Five emerging categories were found in substantial proportions of participants' discourse: “Change in daily practice activities” (98%), “Fragility of previous pedagogical teacher training” (92%), “Enthusiasm and commitment to teaching” (46%), “Overlapping of tasks and work overload” (35%), and “Difficulties in student assessment” (23%). These percentages were obtained using the *NVIVO*® matrix encoding and crosstab tools for searching the association between emerging categories and the speeches of selected participants. We checked for an association between these categories and excerpts from participant speeches in their affiliated departments and found that they were coincident. Table 3 shows the emerging themes, and the respective subcategories identified, with some examples of participants' speeches.

**Table 3:** Thematic and subthemes codification with speech examples for each

Subcategories	Theme 1: Change in daily practice activities
**Change in perspectives about students**	*“…Look at students again within their possibilities and even limitations that some have…”* *“… I started to set aside more time to prepare lessons and always try to place myself in the student’s perspective…”*
**Mode of student assessment**	*“…value feedback even more and apply it in all the possible opportunities. Overall, it was a game changer…”* *“I “relaxed”mores about student assessment. I was motivated to change and th ESMo offered the security and elements for implementation…”*
**Use of new teaching tools**	*“…In the ESMo course, I noticed the opportunities and where application can actually occur, where learning is going to be even better…”* *“… Even my lectures changed (learning objectives are in the first slide).”*
**Reflection about faculty roles**	*“… after ESMo, we go to the classroom much more conscientious. A global view of the process…”* *“…to realize that other things could be done was striking to me, the beginning of a process…”*
**Restructuring of disciplines**	*“…We restructured, and this year we experienced a new restructuring. It is a very innovative discipline…”*
**Sharing peer-to-peer learning**	*“… We did a workshop in leveling [among teachers] and this has been used…”*
	**Theme 2: Fragility in pedagogical formation**
**Lack of formal teaching preparation**	*“… It ends up that learning is “on the fly”. You start teaching, talk to colleagues, learn but nothing formal and we know that there is a whole theory behind it…”* *“… We become teachers and…. And they say that we are teachers, but there is a lot of things to do…or learn…”*
**Need to know new methods and pedagogical tools**	*“… Understand new methodologies, since I only had contact with the traditional methodology in my training…”*
**Need for upgrading or renovating teaching–learning techniques**	*“…I decided to do it [training in HPE] to improve my teaching technique and feedback to the students…”* *“… one of the questions was to learn more, have an in-depth knowledge about teaching methodology, teaching strategies, both theory and practice…”*
	**Theme 3: Teacher engagement and commitment**
**Willingness to improve**	*“… even after so many years, supervising students and everything, I think that it’s never too late to improve, have a better performance and even assess students better…”* *“… We live to learn. It’s always good to learn, see new things and alternatives…”*
**Responsibility for practices**	*“… And since then, the practice of teaching, contact with a content more specialized in a subject can help me improve my classes…”* *“… When possible, whenever there is any teaching formation activity, I am interested…”* *“…I saw something unyielding and felt an internal need, that it could be different, because students nowadays are different…”*
**Enthusiasm for practices**	*“… and more than the student response, what delighted me was the behavior of a student experiencing a technique. Then I really perceived that it’s working out. It’s great…”*
	**Theme 4: Overlapping of functions**
**Complex schedules**	*“…it’s more a matter of dividing between everything you do and finding a time slot not only for face-to-face meetings, but for reading…”*
**Extra-academic activities (research, managment and assistance/extension)**	*“…need to look at teaching. I was very focused on great research projects, even with international partnerships…”* *“…We always find it difficult to set aside some time because of our multitasking…”*
	**Theme 5: “Difficulty in student evaluation processes”**
**Be fair in assessment**	*“…Thinking whether we are coherent, whether the process is fair, whether we are assessing what we hope to assess is difficult…”* *“…We worry about the assessment, assign a value… not only of approval or failure* *decisions, because our profession demands it, doesn’t it?…”*
**Need of improvement and new methods**	*“… assessments that we carry on until now were very simple, weren’t they? So, ESMo opened many horizons, even in terms of applying new assessment methods…”* *“…I needed to slightly improve my assessment methods. It was something much more complicated…”*

## Discussion

In this case study, we observed a great diversity among faculty members and preceptors who participated in the ESMo in HPE. Most have many years of T&L practice, although many reported that they have learned how to teach by doing, with no previous formal training. Accordingly, “*Fragility of pedagogical training for healthcare education*” was the second emerging thematic category detected, which agrees with published studies demonstrating deficiencies in this field during postgraduate programs that theoretically should prepare for teaching [20]. Postgraduate training programs in Brazil are strongly focused on basic or clinical experimental research activities. Thus, many teachers and preceptors working in HPE are faced with expectations for their teaching performance only when they start their careers. This situation leads to established faculty members showing different degrees of effectiveness, job satisfaction, and even permanence in teaching activities, which are all highly dependent on personal effort for training [20, 21, 25]. This gap is also interlinked to the emerging thematic category “*Difficulty in student assessment*” since this process also depends on specific preparation.

In convergence with international studies, we observed immediate positive reactions and satisfaction, together with perceptions of gains in knowledge from the professionals who participated in the ESMo [25, 26]. Qualitative analysis of discourses from interviewed faculty also demonstrated that participation in the ESMo was associated with higher outcomes on the Kirkpatrick model [17], mainly observed in the most frequent thematic category “*Changes in teaching practices*”. Two years after finishing the ESMo, 80% of participants implemented totally (40%) or partially the changes they planned in their intervention proposal. An example in clinical education was the implementation of the mini clinical evaluation exercise (Mini CEx) in the Pediatrics rotation in the Medicine internship. Another example in basic biomedical sciences was the development and implementation of the “virtual microscope” technology for teaching Histology and Cell Biology. In this respect, three participants of the ESMo, with two medical students, had a manuscript accepted in a prestigious journal describing students' perception and academic performance assessed upon virtual microscopy implementation in our school [27]. They had the support and guidance of senior facilitators of our CFD. These are a few examples of how the proposals led to the improvement of medical education and also created scholarship opportunities when faculty members realized that they could have a scientific and innovative approach related to HPE.

The consistent growth of FD initiatives requires supportive institutional leadership, appropriate resource allocation, and institutional recognition of teaching excellence. Relevant results and local changes that lead to educational practice improvement within the institution are the foundation that supports changes [21]. Of note, ESMo participants did not mention either institutional discouragement or policies impeding changes in their daily practices.

Effective participation in FD activities remains a limiting factor in these programs. Resistance to pedagogical changes, overlapping healthcare duties, difficulties related to change management, and work overload related to scientific research and other academic activities are frequently cited as reasons for not participating in FD workshops [28]. We also found this in our study, as demonstrated by one of the thematic categories, “*Overlapping of function*”. Another challenge is that healthcare faculty members, mainly those who graduated in Medicine, consider teaching a secondary, less valued activity [29, 30]. The increment in the number of medical schools in the last ten years in Brazil, comprised mostly of private institutions, and the unofficial reports that the work overload in these new schools may be greater than in the more traditional institutions may increase in a national basis the challenge for Faculty members to dedicate time and attention to FD programs [8]. In this process, the FD axis is often assessed as very fragile or nonexistent.

The “*Commitment and Enthusiasm for Teaching*” category is directly correlated with professional identity. Professional identity should be at the center of FD planning, recognizing the diversity of professionals linked to the institution that contribute to specialized education, thus enabling the construction of a developmental trajectory for each person involved [20, 25].

The workplace should favor this demand and broaden reflection on practices, as well as foster respect for individual differences. Among the goals of our CFD, and directing its approach, is the intention of contributing to building a community of practices and supporting the compounding members on educational practices in diverse professional settings.

Medical and HPE education has evolved with increasing demands on Faculty members who are required to be socially responsible and accountable and to deal with growing pressure for the professionalization of teaching practice. Developing competent professional teachers, educators, researchers, and leaders to new roles and responsibilities in medical education requires FD. This, as McLean (2008) et al. stated, is not an easy task [25]. The lack of opportunities or excessively rigid FD activities could discourage professionals with high potential and enthusiasm for academic activities. Therefore, FD programs focusing on teaching should be flexible [26] and this was done in our program based on the participants´ opinions at the end of each session. The positive and negative comments and, in particular, the suggestions were analyzed by the CFD educators, who planned changes of approach in some contents, such as providing feedback, increasing time on the student assessment, constructing specific new workshops, such as “Good Practices on Item Writing” and “Team-based Learning”. The ESMo for remote education, that we constructed and offered during the Covid-19 pandemic is also an example of the flexibilization and adaptability of our program.

Previous studies indicate that the effectiveness of FD has been associated with its longitudinal and continuous format. It encompasses training for using innovative T&L strategies, effective assessment, and evaluation methods, and reflecting on daily practice in different scenarios, including those comprising the practical work environment where future health professionals training [21]. Steinert (2010) provides an interesting description of how FD activities can move along two dimensions: from individual (independent) experiences to group (collective) learning and from informal to more formal approaches [31]. CFD has been offering the ESMo and on-demand workshops that should be included in the formal group of activities. The CFD also offers informal and individual learning opportunities.

These initiatives are intended to foster and support an educational community of practices within the medical school. Leadership and management skills development have been recognized as critical components for FD in health professions education [30]. In our context, communication between trained faculty members and the CFD is constant and may be carried out on individual demand for one-to-one support, by accessing resources on the CFD´s website [32], or the YouTube channel that we created during the Covid-19 pandemic to support and amplify distance learning opportunities [33].

Steinert (2020) states that to move forward, we need to broaden the scope of FD from teaching to academic development, expand approaches to include peer coaching, workplace learning, and communities of practice, and promote research and scholarship in FD [34]. This is exactly what we attempt to develop in the initiative underlying this pioneering study developed in a traditional medical school in Brazil. We expect that this experience can contribute to and stimulate others at the national level to organize, implement, evaluate, and publicize FD program experiences in the HPE. And doing this, Brazilian authors can contribute to increasing author diversity and reduce the epistemic injustice in the medical education field, as Maggio et al*.* (2022) pointed out in their recent article entitled “*The voices of medical education scholarship: Describing the published landscape*”. They showed that, among 37,263 articles published in 24 medical journals from 2000–2020, only 12,007 (11.4%) were written by Global South authors [35].

This study has some limitations, as FD is not a linear process but a continuum, it may suffer the influence of various factors over time, including external experiences. Non-institutional experiences may also impact educational proficiency. Faculty members who look for FD workshops and agree to participate in qualitative research are usually much more committed to their activities, which may represent a bias.

## Conclusion

This first in-depth evaluation study of the long-term effects of an FD intervention brings the voice of a Brazilian HPE school. The results showed that participation positively changed participants' teaching & learning, and assessment practices. This consistently improved the educational environment, fostering a community of practice and, ultimately, valuing FD processes in the national scenario.

## Data Availability

The datasets used and/or analyzed during the current study are available from the corresponding author upon reasonable request.

## Abbreviations

HPE: Health Professions Education
CFD: Center for Faculty Development
ESMo: Essential Skills Module
FD: Faculty Development
BCG: Brazilian Curricular Guidelines
FAIMER: Foundation for Advancement of International Medical Education and Research
FMRP-USP: Ribeirão Preto Medical School at the University of São Paulo
CFD: Center for Faculty Development
OSCE: Objective structured clinical examination
T&L: Teaching and Learning
VLE: Virtual Learning Environment
FCM: Federal Council of Medicine
SAEME: Accreditation System of Medical Schools
